# ERRATA

**DOI:** 10.6028/jres.092.008

**Published:** 1987-02-01

Corrections to be noted in Volume 91 of the JOURNAL OF RESEARCH of the National Bureau of Standards

**Table:** 

Page	Line	Now reads in part	Should read
82	22, col. 1	[3]	[33]
84	6, col. 1	[36]	[34]
92	Ref. 36	Clin. Chem. **24** 353 (1978).	Clin. Chem. **24** 303 (1978).
167	16, col. 2	$\frac{\text{dI}}{\text{dv}}=K\cdot \log_{e}$	
168	1, col. 1	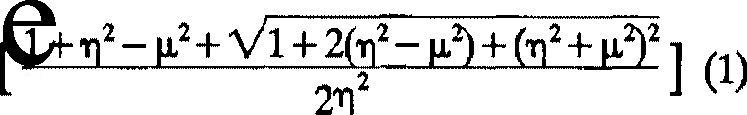	
			$\frac{\text{dI}}{\text{dv}}=K\cdot {\text{log}}_{e}[\frac{1+\eta^{2}-\mu^{2}+\sqrt{1+2(\eta^{2}-\mu^{2})+{\left(\eta^{2}+\mu^{2}\right)}^{2}}}{2\eta^{2}}](1)$
169	7, col. 2	D_water_	Ḋ_water_
175	47, col. 1	T. E. Feuchtwang	T. E. Feuchtwang [22]
197	Eq 3–5	10^6^	10^−6^
200	cap., Fig. 22	61 cm × 25 cm	92 cm × 61 cm × 25 cm
209	fn. 7	and C. F. Quate	S. A. Elrod, A. L. deLozanne and C. F. Quate
211	fn. 9	The author is not aware of any other published results which show…	The author is aware of only one other published result which shows…
217–220			The computer program on p. 217 is correct to the line, “Listing of DNSITY,” one-third down left column, p. 218. Place a 3 at this point, a 2 at “GET WORK FUNCTION,” two-thirds down left column, p. 219, and a 4 at “DIVISION, NATIONAL BUREAU OF STANDARDS,” seventh line from bottom of left column, p. 220. The program should be read in the sequence thus indicated.
289	13, col. 2	The ten are column entries in	The entry in column ten is
296	13, col. 2	are given cellulose	are cellulose
327	14, col. 1	aliquoting	aliquanting
327	54, col. 1	10 °C	110 °C

